# Supplementary material for: TCR microclusters form spatially segregated domains and sequentially assemble in calcium-dependent kinetic steps
Source: Nat Commun. 2019 Jan 17;10:277. doi: 10.1038/s41467-018-08064-2 (PMC6336795; doi:10.1038/s41467-018-08064-2)
Supplement: Supplementary file 1 — Supplementary Information [file 41467_2018_8064_MOESM1_ESM.pdf]

TCR microclusters form spatially segregated domains and sequentially assemble in calcium-dependent kinetic steps

Yi et al.

Supplementary Figure 1

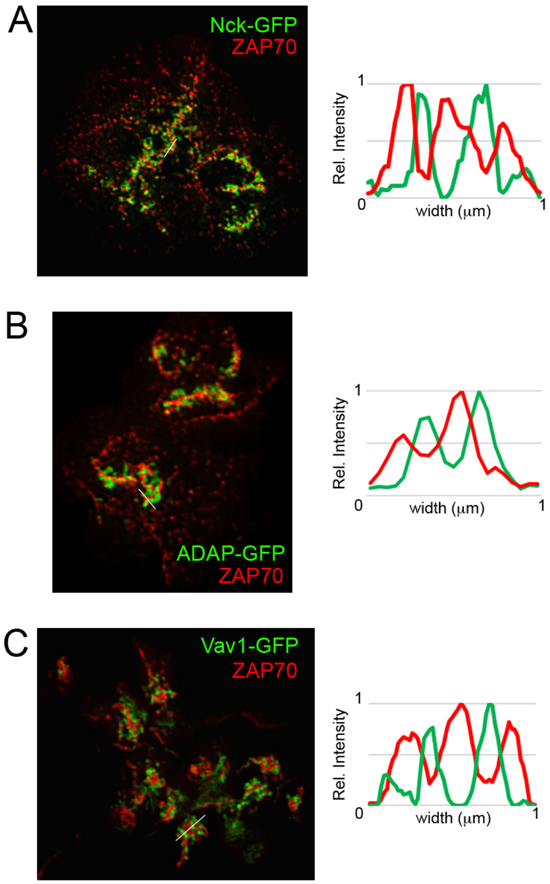

Supplementary Figure 1. TIRF-SIM imaging of microclusters show distinct receptor and signaling domains.
TIRF-SIM images of microclusters formed in Jurkat T cells activated on coverslip-bound anti-CD3 antibody were visualized using A) Nck-GFP (green) and ZAP70-Halo-JF646 (red), B) ADAP-GFP (green) and ZAP70-Halo-JF646 (red), or C) Vav1-GFP (green) and ZAP70-Halo-JF646 (red). Right graph shows relative (rel.) intensity measured across the width of the white line in the corresponding left image.

Supplementary Figure 2

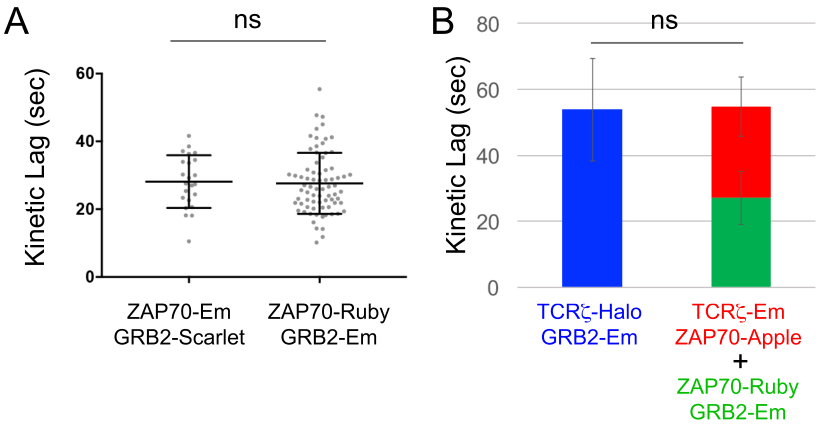

Supplementary Figure 2. Kinetic lags between microcluster components are independent of fluorescent tag.
A) Comparison of average kinetic lag measured between ZAP70-Emerald (Em) and GRB2-Scarlet, and ZAP70-Ruby and GRB2-Em at microclusters in Jurkat cells activated at 21°C. B) Comparison of average kinetic lag measured between TCRζ-Halo-JF646 and GRB2-Em (blue), and the sum of TCRζ-Em and ZAP70-Apple (red) and ZAP70-Ruby and GRB2-Em (green) at microclusters in Jurkat cells activated at 21°C.

Supplementary Figure 3

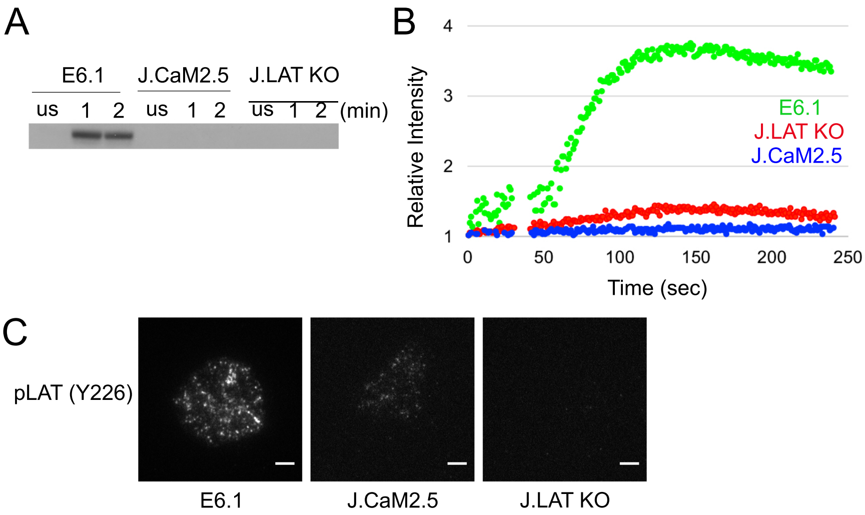

Supplementary Figure 3. Calcium flux is inhibited in J.LAT KO cells.
A) Western blot of lysates from E6.1, JCaM2.5 (LAT deficient), and J.LAT KO (LAT-/-) Jurkat cells detected with anti-pLAT (Y226) antibody in unstimulated cells (us) or at indicated time points following activation with OKT3 antibody. (B) FACS plot showing calcium flux over time of Indo-1 loaded E6.1 (green), JCaM2.5 (blue), and J.LAT KO (red) cells activated with 1 μg/ml OKT3 antibody at 30 seconds. C) E6.1, JCaM2.5, and J.LAT KO cells activated on coverslip-bound anti-CD3 antibody and stained with anti-pLAT (Y226) antibody. Scale bars, 2 μm.

Supplementary Figure 4

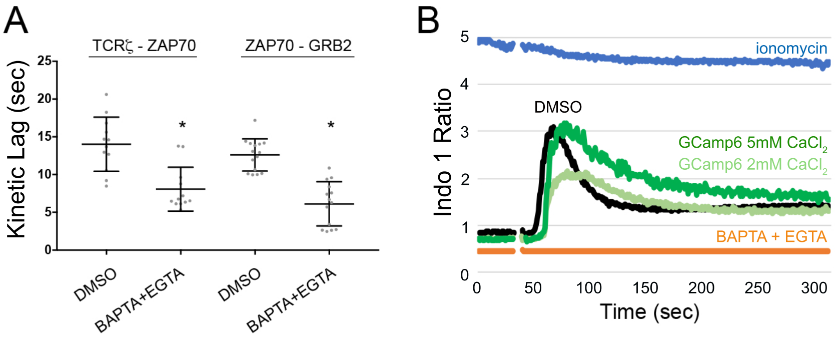

Supplementary Figure 4. Calcium chelation speeds up recruitment of microcluster components at 37°C.

A) Comparison of average kinetic lags measured between TCRζ-Emerald and ZAP70-Scarlet and ZAP70-Emerald and GRB2-Scarlet microclusters in Jurkat cells treated with DMSO or with BAPTA and EGTA and activated at 37°C. B) FACS plot showing calcium flux over time of Indo-1 loaded Jurkat E6.1 cells treated with DMSO (black), BAPTA and EGTA (orange), 1uM ionomycin (blue) and GCamp6 expressing cells either in 2mM$CaCl_2$ (light green) or 5mM $CaCl_2$ solution (dark green). Cells were activated with 1 µg/ml OKT3 antibody at 30 seconds.

Supplementary Figure 5

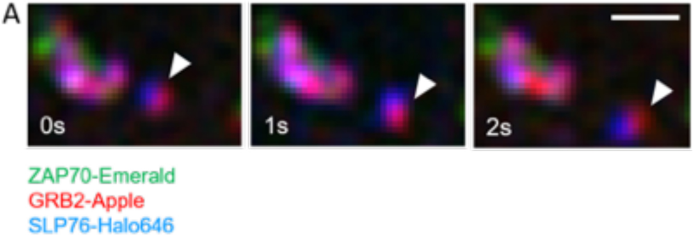

Supplementary Figure 5. Signaling domain clusters detach from microclusters.
A) Time-lapse TIRF-SIM images of ZAP70-Emerald, GRB2-Apple, and SLP76-Halo-JF646 focused on their localization dynamics at a peripheral microcluster. Time-lapse images show a signaling domain cluster marked by GRB2 and SLP76 (white arrowhead) becoming detached and moving away from the microcluster. Scale bar – 1 µM.
