# Supplementary material for: TCR microclusters form spatially segregated domains and sequentially assemble in calcium-dependent kinetic steps
Source: Nat Commun. 2019 Jan 17;10:277. doi: 10.1038/s41467-018-08064-2 (PMC6336795; doi:10.1038/s41467-018-08064-2)
Supplement: Supplementary file 3 — Description of Additional Supplementary Files [file 41467_2018_8064_MOESM3_ESM.pdf]

Description of Additional Supplementary Files

Supplementary Movie 1. TIRF-SIM imaging of microclusters showing delay in recruitment of ZAP70 at TCRζ microclusters. Related to Figure 1A.
TIRF-SIM movie of microclusters formed in a Jurkat T cell expressing TCRζ-Halo646 (red) and ZAP70-Emerald (green) activated on coverslip-bound anti-CD3 antibody. Recorded at one frame every sec, and played back at 10 frames/second.

Supplementary Movie 2. TIRF-SIM imaging of microclusters showing delay in recruitment of LAT to ZAP70 microclusters. Related to Figure 1B.
TIRF-SIM movie of microclusters formed in a Jurkat T cell expressing LAT-Halo646 (red) and ZAP70-Emerald (green) activated on coverslip-bound anti-CD3 antibody. Recorded at one frame every sec, and played back at 10 frames/second.

Supplementary Movie 3. TIRF-SIM imaging of microclusters showing delay in recruitment of SLP-76 to ZAP70 microclusters. Related to Figure 1D.
TIRF-SIM movie of microclusters formed in a Jurkat T cell expressing SLP76-Halo646 (red) and ZAP70-Emerald (green) activated on coverslip-bound anti-CD3 antibody. Insets show zoomed-in views of regions marked by white boxes in the left image. Recorded at one frame every sec, and played back at 10 frames/second.

Supplementary Movie 4. TIRF imaging showing calcium flux following signaling cluster recruitment. Related to Fig. 5A.
TIRF movie of a Jurkat T cell becoming activated on coverslip-bound anti-CD3 antibody at 21°C expressing ZAP70-Apple (yellow), GRB2-Halo-JF646 (magenta), and Fluo-4 (cyan) showing that calcium flux occurs following recruitment of signaling cluster reported by GRB2-Halo-JF646. Recorded at one frame every 3 sec, and played back at 10 frames/second.

Supplementary Movie 5. TIRF imaging showing cell spreading following signaling cluster recruitment. Related to Fig. 5B.
TIRF movie of a Jurkat T cell becoming activated on coverslip-bound anti-CD3 antibody at 21°C expressing TCRζ-Halo646 (red) and GRB2-Emerald (green) showing that cell spreading occurs following recruitment of signaling cluster reported by GRB2-Emerald. Recorded at one frame every 3 sec, and played back at 10 frames/second.

Supplementary Movie 6. TIRF-SIM imaging showing signaling domain clusters detach from microclusters. Related to Supp. Fig. 5.
TIRF-SIM movie of a Jurkat T cell becoming activated on coverslip-bound anti-CD3 antibody expressing ZAP70-Emerald (green), Grb2-Apple (red) and SLP76-Halo646 (blue) showing signaling domain clusters marked by GRB2 and SLP-76 become detached and move away from the microcluster marked by ZAP70. Recorded at one frame every second, and played back at 10 frames/second.
